# The Odyssey’s mythological network

**DOI:** 10.1371/journal.pone.0200703

**Published:** 2018-07-30

**Authors:** Pedro Jeferson Miranda, Murilo Silva Baptista, Sandro Ely de Souza Pinto

**Affiliations:** 1 Department of Physics, State University of de Ponta Grossa, Paraná, Brazil; 2 Institute for Complex System and Mathematical Biology, SUPA, University of Aberdeen, Aberdeen, United Kingdom; University of Texas at Austin, UNITED STATES

## Abstract

In this work, we study the mythological network of Odyssey of Homer. We use ordinary statistical quantifiers in order to classify the network as real or fictional. We also introduce an analysis of communities which allows us to see how network properties shall emerge. We found that Odyssey can be classified both as real and fictional network. This statement is supported as far as mythological characters are removed, which results in a network with real properties. The community analysis indicated to us that there is a power-law relationship based on the max degree of each community. These results allow us to conclude that Odyssey might be an amalgam of myth and of historical facts, with communities playing a central role.

## Introduction

The paradigm’s shift from reductionism to holism stands for a stepping stone that is taking researcher’s interests to the interdisciplinary approach. This process is accomplished as far as the fundamental concepts of complex network theory are applied to problems that may arise from many areas of study. Such areas include physics, social sciences, communication, economy, financial market, computer science, internet, World Wide Web, transportation, electric power distribution, molecular biology, ecology, neuroscience, linguistics, climate networks and so on [1, 2]. As the study of objects advances under the network’s paradigm, some classes of networks arise as a function of their statistical structures [3, 4]. Given these structures, some patterns can be associated with networks measures which determine their classification [5].

Introducing these concepts to the social sciences implies that we can build social networks from sets of observable sociological relations, such as individual interactions, associations of human groups, Internet’s social networks, etc. The repertoire available of statistical measures of social networks increases as far as this procedure is applied to different cases. In this manner, one can define a sort of taxonomy of social networks that can be built by the simple comparison of their statistical properties [6]. Beyond statistical properties, such social analysis also allows deep predictions, like human activity patterns over a spatial layout [7]. With conformity with this reasoning, we appeal to the statistical universality, using it to unify social network into characteristic groups [8].

A priori, social networks can be extracted from any narrative that contains descriptions of social relationships. This procedure can be done if some standard criteria are met to avoid arbitrary tendencies [9]. As far as some narratives might contain uncertain historicity, so will be their resulting social networks. To address this problem, we rely on the concept of statistical universality to classify networks based on narrative into characteristic groups. This classification is made by means of topological measures that generate statistical properties which allow us to determine the class of networks.

In this work, we are especially interested in two classes of social networks: real networks and fictional networks. Real social networks are small world [4, 10], hierarchically organized [11, 12], highly clustered, assortatively mixed by degree [13, 14], and scale-free [4, 15]. Additionally, since real networks are scale-free, its degree distribution follows a power law [4]. They also possess giant component with less than 90% of the total number of vertices and are vulnerable to targeted attacks while robust to random attacks [16].

On the other hand, we have the fictional social networks, which can be characterized as being small world, feature hierarchical structure; exponential law dependence of degree distribution, implying that it is not scale-free. Moreover, it holds giant component’s size larger than 90% of the total vertices, shows disassortativity by degree, and is both robust to random and targeted attacks [17, 18]. These last references are related to Marvel Universe’s fictional network. To increase the examples of networks based on fictional networks, we list the networks studied by Carron and Kenna: Les Miserable, Richard III, The Lord of the Rings: The Fellowship of the Rings, and Harry Potter [19]. These networks have similar properties of that presented in [17] and [18].

This list of statistical properties of general networks enables us to classify most networks based on narratives as real or fictional. In a similar arguing, a pioneer study concerning three mythological narratives with uncertain historicity was made for Beowulf, Iliad and Táin Bó Cuailnge [19]. Topological quantifiers were collected, and their final conclusions demonstrated that Iliad’s social network behaves most like a real social network. The same could be acceptable for Beowulf and Táin Bó Cuailnge if some convenient topological modifications are carried out. This work opens a new way to serve comparative mythology in order to assess the historicity of a given mythological narrative.

On the work with Iliad of Homer, Carron and Kenna [19] cited that it has historical justification since there are some recent archeological findings that support some of its events [20, 21], supporting their affirmation that Iliad can be based on real historical facts. Inspired by their work, we wonder how the mythological presence of beings such as gods, heroes, and magical beasts affect the underlying social network. Does it imply some interpretation that gives rise to mythological personifications or it is just an allegory inside historical data? These questions instigate us to think if Homer wrote his poems mostly based on his experience in the local society picturing normal, but influential people as mythological characters; or if it is a picture of the local society mixed with an invented mythological drama.

Keeping this questioning in mind, and considering their relevant results of Iliad’s social network, we propose a new study for the Homer’s Odyssey [22–24]. For this, we will be considering the main statistical properties of networks and the underlying communities of Odyssey’s mythological network. Communities are dense fractions of the network, which encompasses dense relations of a given set of vertices. The presence of communities is expected in real social networks [25], more importantly, its structure provides us with insights into the intricate influential relationships between the mythological and normal characters. We argue that communities’ analysis is fundamental to understand how social cliques are associated with each other. It also gives us a clue of how mythological and human characters are organized into communities. In this manner, we wish to find the clues that explain the communities of Odyssey.

As a real network reference for our model, we use a Facebook posts network. In a recent study for a Facebook “friendship” network, for one hundred colleges and universities, they managed to detect and examine communities concluding that the statistical study of microscopic (*i*. *e*., local relations, and social background) and macroscopic (*i*. *e*., network partitions, and communities) aspects of a social network are complementary [26]. It is worth noting that our choice for using such network as a criterion of “realness” of Odyssey’s network is based on two main reasons: the first stands for our access to a large data of human interactions by means of posts, this feature allows us to have a good basis for statistical comparison, since large data assembly implies in stabilization of statistical quantities of social networks; the second stands for the similarity between our build criteria and the Facebook’s post network. In addition, on Facebook, most people tend to build their personal profile and friendship based on their real-life social circles [27].

Given this background, the main objective of this work is to study the social mythological network of Odyssey of Homer by means of known social network analysis and also by means of community analysis.

## Methodology

### Description of the narrative

Along with Iliad, the Odyssey of Homer encompasses most of the Mythology’s background of the Western civilization. These epics date around the VIII century B.C., after the writing system development using the Phoenician alphabet [23, 24]. It is known that Odyssey relates to some echoes from the Trojan War narrated mainly on Iliad. The study realized with Iliad’s network showed that it satisfied most of the statistical properties expected in real networks. Additionally, archeological evidence was found in the region of Anatolia which supports the historicity of some conflict occurred during the XII century B.C. This finding is an indication that the conflict between Greeks and Trojans possibly occurred [20, 21]. As a continuum of Iliad, the Odyssey tells the tale of Odysseus’s misadventures on his struggle for returning to his homeland. This saga takes ten years long until Odysseus reaches Ithaca after the ten years of warring on the Trojan War. The epic poem has 24 chants in hexameter verses, where the tale begins ten years after the War in which Odysseus fought along with the Greeks.

The structure of the text is composed of four main parts. Firstly we have the chants I, II, III, and IV, “Assembly of the gods”. The second part covers the chants V to VIII, “The new assembly of the gods”. The chants IX to XII constitutes the third part, “Odysseus’s Narrative”. Finally, in the fourth part, we have the “Journey back home” in the chants XIII to XXIV. Odyssey is considered to be a masterwork in Western Literature, for it holds a set of adventures often considered more complex than Iliad; it has many epic aspects that are close to human nature, while the predominant aspect of Iliad is to be heroic, legendary and of godlike wonders. However, there is a consensus that Odyssey completes Iliad’s picture of the Greek’s mythology. These two poems together prove Homer’s geniality and universality, being both of fundamental importance to universal poetry in the ancient occident [23].

### Data assembly

Odyssey’s poem is composed of an unusual vocabulary, making it difficult to identify a character only by her name. It turns out that some of them are referenced by some unique title, which most of the time reflects her deeds in the story. For example: “…Tell me, O muse, of that ingenious hero who traveled far …” this ingenious hero is actually Odysseus; “Then Athena said, "Father, son of Chrono, King of kings …”“ we find out that Athena is talking to Zeus. Given this example, there is a necessity to adopt a careful text analysis to identify all relationships between characters. This nuance generates impossibility to an automatic extraction of the network structure [9], so we propose a method to build the network based on textual interpretation. It is worth citing that Carron and Kenna [19] built their mythological network using three social interactions: friendly, hostile and both. Their building criteria differ from ours in a sense that we are to explain.

First, to eliminate subjectivity in the construction of the network, we created a non-arbitrary building process. In social networks derived from narratives, vertices represent characters and edges represent at least one form of social interaction between them. Thus, we affirm that an interaction exists, if some of the following conditions are met:

1. *The criterion of the shared event*: characters are in the same place at same time, showing to participate in the present action.
2. *The criterion of conversation*: characters speak to each other directly;
3. *The criterion of indirect relation*: characters cite one another to a third. This criterion shall prevail if, in the act of the citing, the interlocutor describes the cited with minimal information beyond her name.

**Fig 1:**
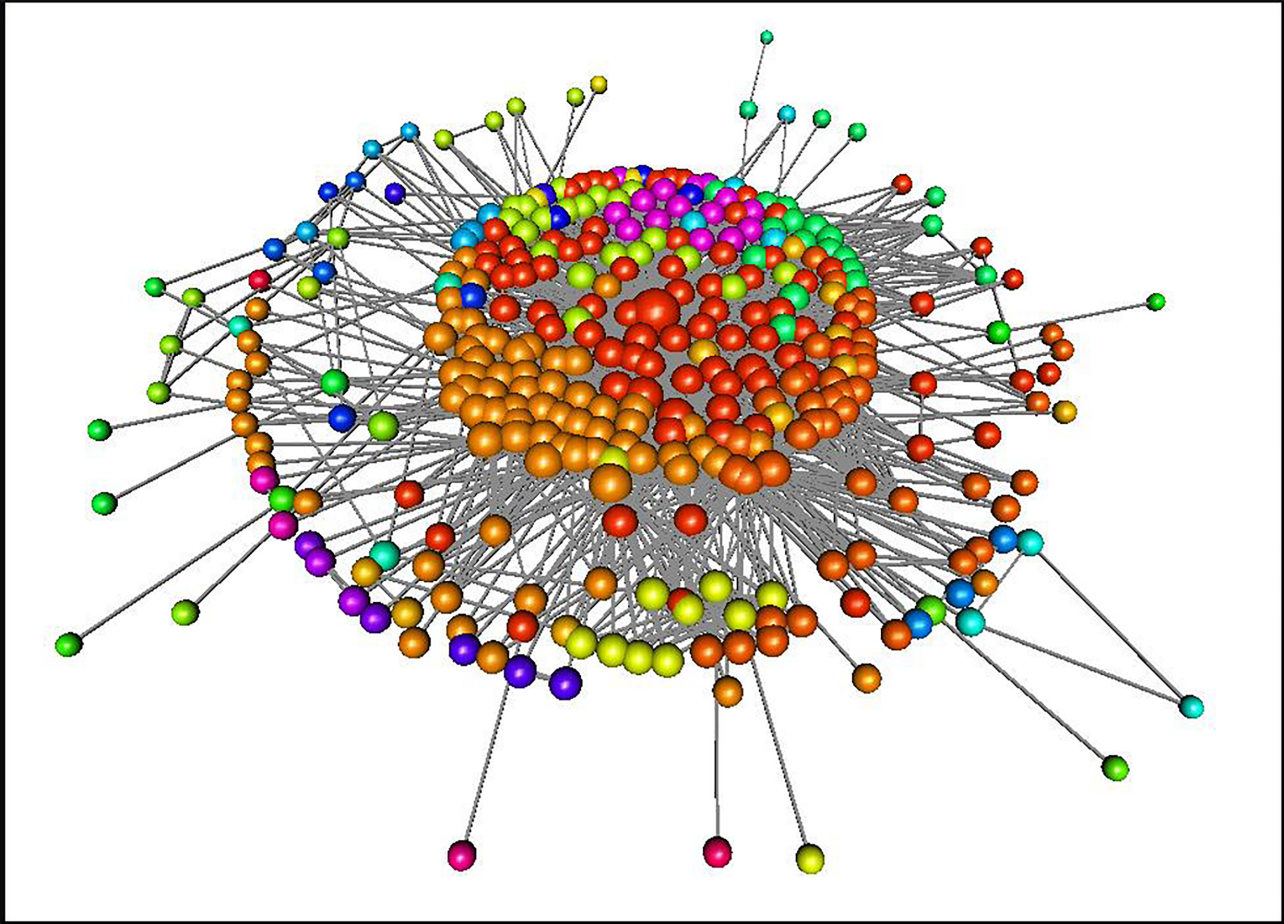
Odyssey’s mythological network. The coloring of the vertices is associated with the variety of communities. The vertex size is based on its topological importance in the network.

To avoid some possible misleading interpretation of the narrative’s social relations, we studied different editions and revisions of Odyssey [22–24]. We realized that the basic differences from the Odyssey’s translations produced no significant deviation in the network creation process. The building criteria allowed us to identify 342 unique characters linked socially by 1747 relations (Fig 1). We also point out that this network is socially limited, for it is based on the events limited in the narrative.

### Network measures methods

Our method to study Odyssey is based on two fronts: an ordinary statistical analysis and the analysis of communities. We defend that the analysis of communities complements the statistical analysis, such that both can enhance the accuracy in the determination of Odyssey’s classification. Statistical analysis must measure: “small-worldness”, network clustering, the fitting of power-law degree distribution, the scale-free phenomenon, hierarchical by vertex, assortativity by degree, the size of the giant component, and the tolerance to random and targeted attacks. The computation of all these measures allows to statistically classifying Odyssey’s network into a class of social network.

The network will be small world if *ℓ* ≈ *ℓ*_*rand*_ and *C* ≫ *C*_*rand*_ are both satisfied [10]. Where *ℓ* is the average path length and *ℓ*_*rand*_ is the random average path length of a random network built with the same degree distribution. Such that *C* stand for the clustering coefficient, and *C*_*rand*_ for the random clustering coefficient of a random network built with same degree distribution [8]. Both real and fictional social networks are small-world. As an accessorial measure, we also use the diameter *ℓ*_*max*_ of the network that stands for the largest geodesic within it.

Thus, the network will be considered highly clustered if *C* has values close to 1. When Albert and Barabási describes the measure of *C*_*i*_, it is a local value computed to each vertex. This means that *C* is a mean that stand for the tendency of the whole network to possess clusters [8].

Each vertex will have a certain number of edges that make the connection to other vertices; this will be the degree *k* of the vertex. The averaging over all degrees gives us the mean degree 〈*k*〉 of the network. Collecting the degree of each vertex, we can count the relative frequency for k, that is *p*(*k*) (*i*. *e*., the probability of finding a vertex with degree *k*). The plot of *k* versus *p*(*k*) is the degree distribution [8]. For many real network, its degre,e distribution can be expressed as

$$p(k)∼k^{-\gamma },$$ (1)

for a positive and constant *γ* [8]. It configures the power law dependency of degree distribution which is known to be a feature of many real networks; this is an indication that the network is scale-free [5, 19]. In terms of graph theory, that sort of network is composed of a few vertices with high degree, and many vertices with a low degree [15]. On the other hand, the fictional social networks have degree distribution following an exponential decay [19].

Additionally, we also use a supplementary model to describe the degree distribution: the power-law with an exponential cut-off [28]. This distribution is given by the following fitting

$$p\left(k,\alpha ,\beta ,\gamma ,\nu \right)=\nu \frac{e^{-k\beta }}{{\left(k+\alpha \right)}^{\gamma }},$$ (2)

where *k* is the degree, *ν* a normalization parameter of the distribution, *β* a parameter of anomaly adjustment to vertices with high degree from the “tail” of the distribution, *α* a parameter of anomaly adjustment for low degree vertices and *γ* a parameter of the exponential decay cut-off law [28]. A degree distribution fitted according to Eq. (2) is characterized by a “head” following a power law and a “tail” following an exponential law.

Both real and fictional networks show a modular structure that is equivalent to say that the network is hierarchical by vertex. This implies that small groups of vertices combine between themselves into increasingly larger groups. We can test the presence of hierarchy by vertex in a network by finding a strong fit for the power-law dependence of the averaged clustering coefficient versus degree [11, 12]:

$$\langle C(k)\rangle ∼\frac{1}{k}.$$ (3)

To measure the assortativity by degree, we simply apply the Pearson Correlation *r* for all pairs of *N* vertices from the network. For positive values of *r*, implies a tendency of vertices with the same degree to be attached to each other; for negative values of *r*, implies a tendency of vertices to attach to vertices with different degree. In the theory of social network, many real social networks have positive *r* [13, 14], and fictional social networks have negatives values of *r* [17, 19].

The size of the giant component *G*_*c*_ is the subset of the set of vertices from the graph that implies in maximum connectedness [8]. Roughly speaking, real social networks usually have giant component’s size smaller than 90%, while fictional social networks have larger than 90% [16, 19]. There is evidence that scientific collaboration networks are more likely to bear giant component size between 80 and 90% [29]. In particular, this investigation carried out by Newman (2001) showed that out of seven observed collaboration network, only one displayed giant component size of 92.6%, which is still close to the expected 90% value. In a similar study with collaboration networks, Guimerà *et al*. (2005) showed that out of five networks, one displayed giant component size of 92% [30]. Thereby, it is expected that most of these real based collaboration networks should display giant component smaller than 90%. Since this feature is not a universal criterion, it is not mandatory for real social network, but can still be considered a reliable feature considering other statistical attributes.

To test the attack’s tolerance of the network, we first consider a measure of importance for vertices. One of the most complete measures of importance is the betweenness centrality [8]. This measure takes into account simultaneously the degree and the number of geodesics that passes through it. If we consider *σ*(*i*,*j*) as the number of paths between vertices *i* and *j*, and number of these paths that cross a given vertex *l* is *σ*_*l*_(*i*,*j*), such that the betweenness centrality of *l* is

$$g_{l}=\frac{2}{\left(N-1)(N-2\right)}\sum_{i\neq j}\frac{\sigma_{l}(i,j)}{\sigma (i,j)}.$$ (4)

With betweenness centrality, we can calculate the importance of all vertices and rank them in a decreasing order. The first elements of this ranking are the most relevant vertices, and their removal defines the targeted attack. The level of tolerance measured by this attack is observed on the behavior of the giant component: if the giant component reduces drastically as we perform this attack, we say that it is vulnerable to targeted attacks. If the giant component doesn’t decrease drastically, we say that it is resilient to targeted attacks [8, 31]. Note that, since real social network is mostly scale-free, the removal of important vertices causes drastic decrease on the giant component. This implies that real social networks are very vulnerable to targeted attacks, while fictional ones are resilient to them [16, 19]. Conversely, random attacks are defined by the removal of vertices independent of their importance. Then both real and fictional social networks are resilient to random attacks.

As we propose an analysis of communities, it is necessary to define community in terms of graph theory. This concept may not be very well defined formally since there are many valid definitions to it. In general, they are too restrictive or cannot be computed efficiently. But a consensus can be reached if we consider that a partition *P* = {*C*_1_,…*C*_*k*_} of the vertices of a graph *G* = (*V*,*E*), (∀*C*_*i*_ ⊆ *V*) represents a restriction to the community structure, it is expected that the density of internal edges is high compared to the density of edges between them. There are a number of methods to compute the community structure *P*, most of them are based mainly on two criteria: a quality function definition that measures the significance of the community structure, and the algorithm that is used to optimize such quality function [32]. In sum, there is evidence that a community analysis is a powerful tool for real network, which can simplify considerably the network analysis [33]. Specially, in social network investigations, the determination of the community structure has broad applications, since vertices that compose communities tend to have mutual properties [32]. In the Odyssey’s network, we will see that communities are composed by characters that play roles in common events throughout the narrative. In addition, our community analysis heavily relies on the detection of the community structure, ergo on its method.

In this paper, we are not interested in developing a community detection method for our own, but it is necessary to choose one method to detect communities that generate a reasonable meaning in the case of Odyssey. If we look at the building criteria that we have devised to analyze Odyssey, it is notable that sharing events is central in this study. That is, the second and third criteria are special cases to cover all possibilities based on criterion one. We choose such approach because Odyssey is written focusing on events, dialogs, colloquies, fights, disputes, feasts, storytelling, banquets, and so on. Thus, we must utilize a method to compute such communities in order to perceive naturally these events in the network.

It is known that there is a meaningful importance of the community structure in social networks investigations [34]. Moreover, there are some studies that show correlations between community structures and dynamical systems, such as synchronization [35] and a wealth of studies comprising random walks [36–42]. Particularly, Pons and Latapy (2006) showed that the computation of the community structure in large networks has consistent results using random walks [36]. They proposed an algorithm coined as *Walktrap* for this end. Their procedure is based on the tendency of walkers to get “trapped” into densely connected partitions of the network. Considering that *n* is the number of vertices and *m* the number of edges, in the most difficult cases–*i*. *e*., highly dense graphs–, the Walktrap Algorithm runs in time $O(mn^{2})$ and space $O(n^{2})$. For most real cases, the algorithm runs in time O(mlogn) and space $O(n^{2})$. Additionally, the authors have tested their algorithm and compared the results in terms of the quality of the communities, time and size of the network. The comparison was made with the following methods: Gilvan Newman Algorithm, Fast Algorithm, Donetti and Muñoz Algorithm, Netwalk Algorithm, Markov Cluster Algorithm, Düch Arenas Algorithm, and the Cosmoweb Algorithm [36]. The quality function that they utilized was Rand [43] index improved by Hubert and Arabie [44].

The tests that they carried out showed overall high-quality communities as the size of the network increased. The test of time showed that their method is regular, that is, there are more “cheaper” tests in terms of computation. Furthermore, the author also compared the steadiness of Rand index versus modularity for partitions size varying from 100 to 30000: the results showed that their method was slightly steadier than the other methods [36]. It is well known that modularity is the main quality function utilized in method to detect community structures [32]. Additionally, in a recent comparative study of community detection methods utilizing NMI parameter [45], Zhao *et al*. have showed that for network with mixing parameter *μ* ≤ 0.5 and size *N* ≤ 1000 (which is the case of Odyssey’s network), most of the studied methods gives accurate results and are computational affordable, including the Walktrap Algorithm. Considering these facts and the nature of our work, we utilized the Walktrap algorithm to identify the community structure of Odyssey’s network.

After detecting the communities, we compute the statistical analysis to each of the most relevant communities with *N* ≥ 10. This procedure allows us to look at each community as an isolated social network and understand how it behaves. Still, we can also understand how the combination of communities can determine the behaviour of the whole network. In particular, one fundamental concept that we intend to analyse is of the leaders of communities’ [34, 46]. Specially, for Odyssey’s network, a leader will be considered as a character that is almost always present in the set of events comprised within a community. In other words, a leader of a community is a vertex that has the higher degree value in it. When the defined leaders are found, we propose to test some distribution to see if they behave in some known pattern.

## Results and discussion

### Statistical results

A summary of the observed topological properties is compiled, along with Carron’s & Kenna’s results for their mythological networks [19], in Table 1.

**Table 1:** Summary of topological properties.

Network	*N*	*E*	〈*k*〉	*ℓ*	*ℓ*_*rand*_	*ℓ*_*max*_	*C*	*C*_*rand*_	*G*_*c*_	*r*
*Odyssey*	342	1747	10.21	2.58	2.75	6	0.28	0.11	342 (100%)	-0.15
Odyssey*	318	1129	7.10	4.08	3.10	11	0.54	0.06	274 (86%)	0.09
*Iliad* ^*a*^	716	-	7.40	3.54	3.28	11	0.57	0.01	707 (98.7%)	-0.08
*Beowulf* ^*a*^	74	-	4.45	2.37	2.88	6	0.69	0.06	50 (67.5%)	-0.10
*Táin* ^*a*^	404	-	2.76	2.76	3.32	7	0.82	0.02	398 (98.5%)	-0.33
Beowulf*^a^	67	-	3.49	2.83	3.36	7	0.68	0.05	43 (64.2%)	0.01
Táin*^a^	324	-	3.71	3.88	4.41	8	0.69	0.01	201 (62%)	0.04

Size (N), number of edges (E), average path length (ℓ), diameter (ℓmax), clustering coefficient (C), size of the giant (G_c_) and assortativity (r). Odyssey*, Beowulf* and Táin* are the same original network plus some character modification.

^a^ information gathered from [19].

Odyssey’s network has average path length similar to its associated random network. Moreover, it has high clustering coefficient compared to its random clustering coefficient, indicating that Odyssey is small-world. The “small-worldness” feature is expected in both real and fictional social networks. However, in the next section, we explain this result more precisely.

Note that the observed mean clustering coefficient is 0.28, which implies that Odyssey is not as highly clustered as Iliad, Beowulf as well as Táin (Table 1). We revisit this measure later in this section and discuss its nature.

**Fig 2:**
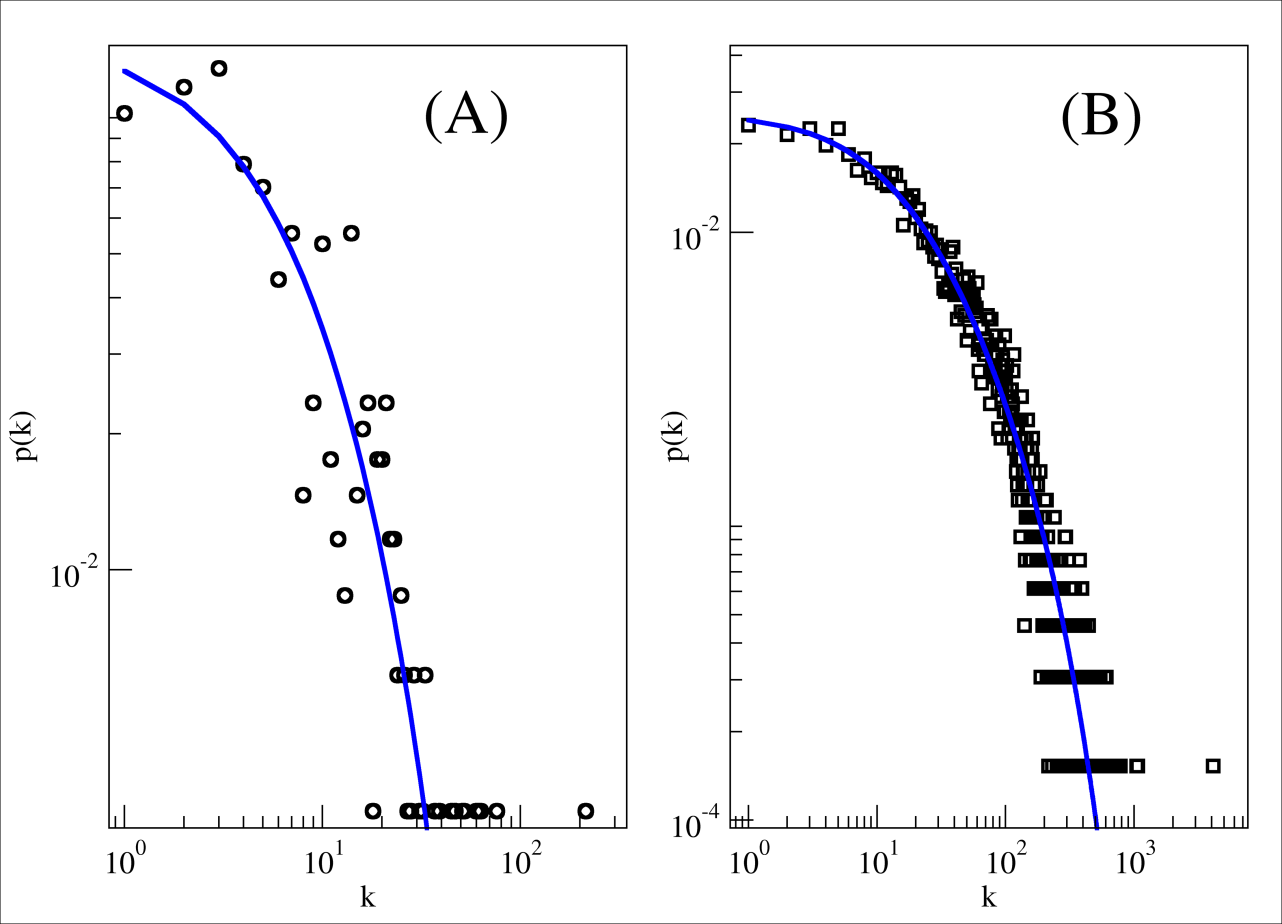
Log-Log degree distribution for Odyssey and Facebook. (A) Odyssey with power-law with an exponential cut-off, and (B) Facebook with power-law with an exponential cut-off. The squared Pearson coefficient for (A) is *R*^2^ = 0.92 and for (B) is *R*^2^ = 0.99.

The degree distribution follows a power law dependence for *γ* = 1.2 ± 0.1 (with *χ*^2^/*df* = 0.06) and *R*^2^ = 0.77, showing that the network behavior is partially scale-free network. However, when we tested a power-law with an exponential cut-off we obtained a better fitting *χ*^2^/*df* = 0.01 (Fig 2). We also applied the same fitting for the real Facebook’s posts network, in order to justify this sort of fitting in social networks.

Facebook’s network has over 63 thousand vertices, which represents people and above 1.3 million edges, which represents posts that connect people [26]. Then we compared both distributions and fitting in order to find out how much Odyssey and Facebook resembles each other in terms of the degree distribution. These plotting and fittings can be found in the Fig 2, where item (A) stands for the power-law with an exponential cut-off for the Odyssey and item (B) for the power-law with an exponential cut-off for the Facebook. The parameters of the fitting in (A) are *α* = 8.76, *β* = 0.08, *γ* = 0.84, *ν* = 0.91, and *R*^2^ = 0.92. And the parameters of the fitting in (B) are: *α* = 2.87, *β* = 0.005, *γ* = 1.03, *ν* = 0.32, and *R*^2^ = 0.99.

This result indicates that the fit for the power law with exponential cut-off is meaningful both for a real network and mythological network. This result is different from what we have studied since now since it contradicts the fit for degree distribution both for real and fictional networks. Overall, we can still affirm that this fit does not exclude the scale-free feature, since the “head” of the distribution continues to be a power-law distribution, signaling a real network. However, its “tail” is still exponential which signals a fictional network. We shall retain the explanation to solve this apparent paradox until the next section.

**Fig 3:**
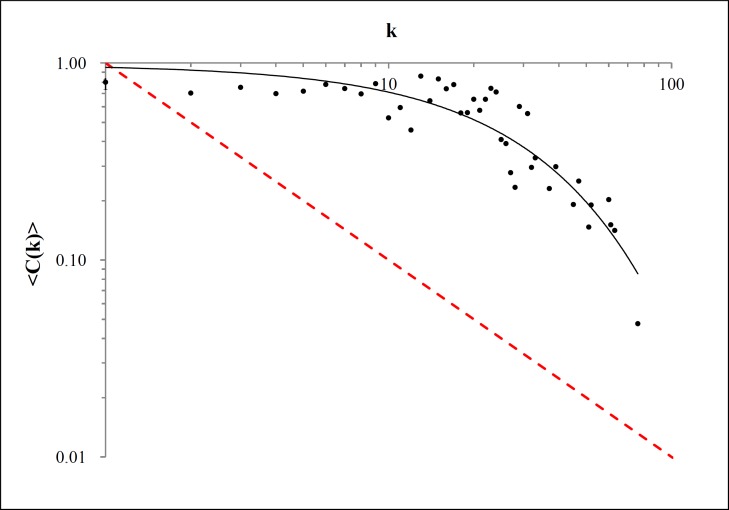
Mean clustering coefficient versus degree. The red dashed line holds for the power law 1/k and the black fit curve for exponential decay with ***R***^**2**^ **= 0.83**.

Odyssey is not hierarchical by vertex since the plot displayed in Fig 3 does not follow a fit for Eq. (3). This result instigates us to test a better fit to those points. Thus, the best fit for this data was an exponential decay law, given by the following equation

$$\langle C(k)\rangle =\mu .e^{-\gamma k}.$$ (5)

The black tendency line displayed in Fig 3 indicates to us that for *μ* = 0.98 and *γ* = 0.032 the exponential decay (Eq. (5)) fits for *R*^2^ = 0.83. The power-law fit would provide a *R*^2^ = 0.52, which we consider a weak fit. The law of hierarchy is also contradictive to what we have seemed until now, because both real and fictional network are expected to be hierarchical. Again, we will revisit this result on the next section.

Odyssey is disassortative by degree, a feature observed in fictional social networks. This means that there is a tendency of vertices with high degree to be connected to vertices with low degree. According to Carron and Kenna, disassortativity may reflect conflicts involving the protagonist in the narrative and was observed in Táin and Beowulf [19]. To overcome this bias, we have tested the impact on assortativity as a consequence of the removal of mythological characters from Odyssey. These results can be found in Table 2. We note that disassortativity is very sensible to the removal of those characters. This means that Odyssey’s disassortativity is due to the presence of some mythological characters that interact frequently with low degree characters. This is especially true since many characters are only cited when at the presence of gods, and this explains such disassortativity. Table 2 also shows us the impact on the giant component, and this indicates that its size is dependent on the presence of mythological characters. This is also a result to be discussed in the community analysis.

**Table 2:** Odyssey’s network main characters removal along with assortativity and giant component responses.

Character	Assortativity	Size of the giant component
Complete network	-0.15	100%
Odysseus removal	-0.07	97%
plus Zeus removal	-0.06	97%
plus Telemachus removal	-0.03	95%
plus Athena removal	-0.04	93%
plus Penelope removal	-0.04	92%
plus Menelaus removal	0.007	92%
plus Hades removal	0.03	92%
plus Poseidon removal	0.06	91%
plus Persephone removal	0.09	86%

The observed giant component in Odyssey contains all the vertices of the network, suggesting two possible causes: there is actually no giant component in Odyssey’s network, or the information contained in the Odyssey’s narrative is not enough in order to display the giant component. A giant component’s size larger than 90% indicates, most probably, that the network is fictional. However, Table 2 has shown us that the giant component’s size is very dependent on the presence of mythological characters. That is, if we disregard mythological characters, the network will mostly behave like a real network.

Thus, the directed attack showed that the network is vulnerable to it while being resilient to random attacks (Table 3). Vulnerability to targeted attack and resilience to random attacks indicate that the network is real.

**Table 3:** Targeted and random attacks.

Targeted Attack		Random Attack	
*G*_*c*_		*G*_*c*_	
No attack	342 (100%)	No attack	342 (100%)
5%	274 (79.6%)	5%	332 (93.6%)
10%	188 (54.6%)	10%	309 (89.9%)
15%	163 (47.3%)	15%	282 (81.9%)
20%	121 (35.1%)	20%	273(79.3%)
25%	41 (11.9%)	25%	248 (72%)

It is displayed the size of giant component (*G*_*c*_) response in terms of absolute and relative impact.

We notice that the removal of central characters causes the network to behave much like a real social network. That is, it becomes assortatively mixed by degree and has the size of the giant component smaller than 90%. Besides giant component and assortativity, it is also detected additional modifications after the removal of most central characters that happens to be the mythological characters. These additional modifications are collected under Odyssey* in Table 1. The difference between the average path length and the random network average path length increased. This implies that the network lost some of its small world pattern with this modification. However, the clustering coefficient’s difference is enhanced, compensating the average path length difference. In general, the removal some mythological characters cause the network to lose some of its short-cuts.

An observable fact is a similarity in the values of the mean degree and the diameter of Iliad and Odyssey*. After the removals, the analyzed Odyssey’s characters network becomes closer to the Iliad’s network, something worth noting since Iliad was rendered real based [19]. Additionally, the degree distribution shows no significant difference after the removal.

These results imply that the Odyssey’s network can be perceived as an amalgam of fictional and real aspects. We can infer that fictional effects can be built up by fictional characters (heroes, gods, and monsters) that form a steady structure for the whole network where the remaining characters can attach. We will discuss this possibility in the community analysis.

The statistical analysis gives us support to affirm that Odyssey is small-world, not hierarchical and semi scale-free. It also has a giant component bigger than 90% and is disassortative. Additionally, we can say that it is resilient to random attack and vulnerable o targeted attacks. As we have seen, the distinguishing features of fictional networks are exponential degree distribution which implies that it is not scale-free, giant component bigger than 90%, robust to targeted attacks and disassortative.

Until now we have shown that the giant component’s size and the disassortativity are dependent on mythological characters, which indicates to us that these characters can cause these features. This also corroborates to Carron and Kenna results when the same approach are performed [19]. Still, we need to know how this fitting for the degree distribution occurs, and how it can be analyzed. Additionally, both real and fictional network is hierarchical, but Odyssey’s network isn’t. To answer all these questions we propose the community analysis.

### Community analysis

**Fig 4:**
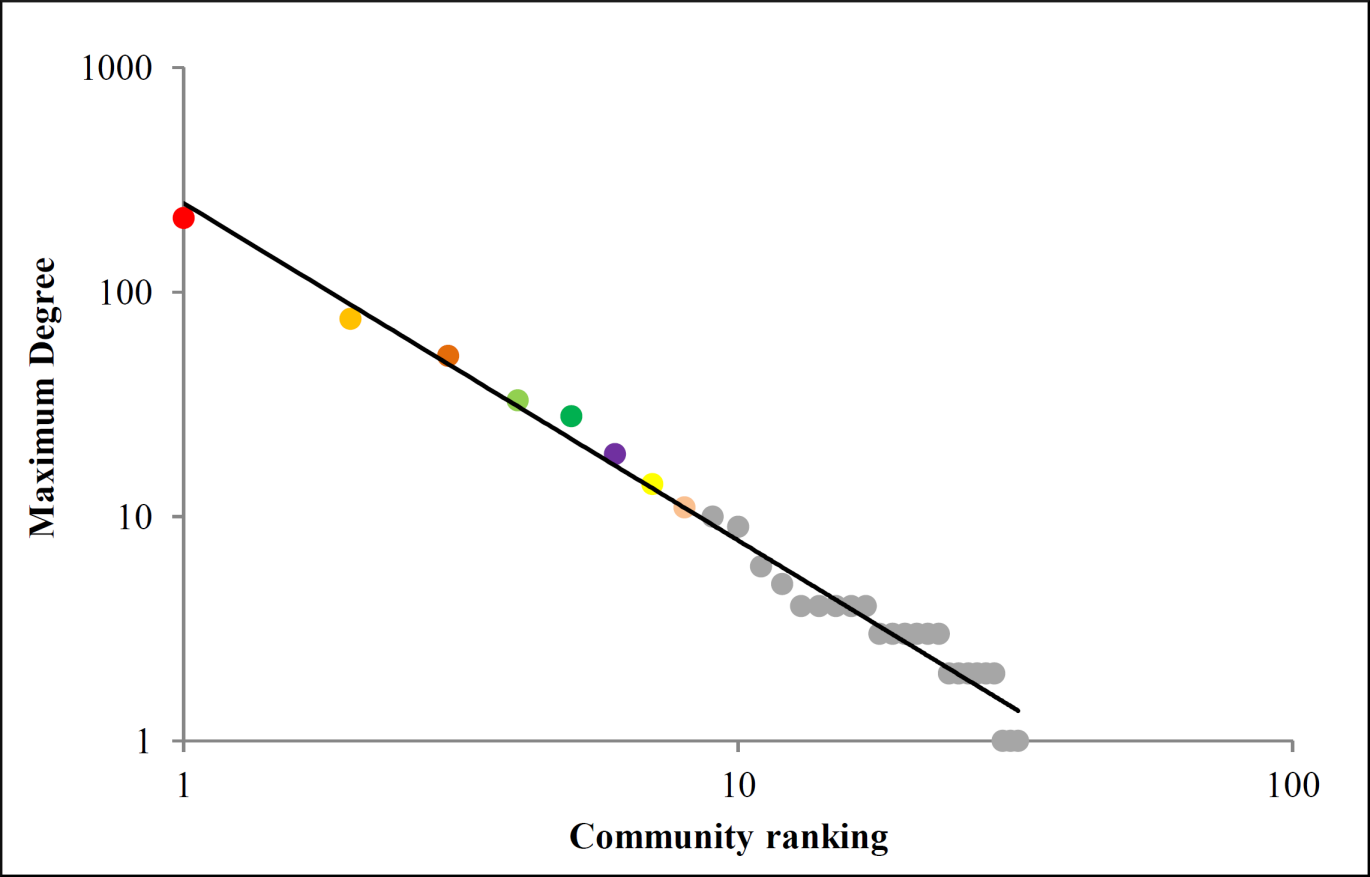
Leaders of communities’ versus community’s descending ranking. The black line is the power law fit with R^2^ = 0.98. The colorings of the dots are associated with each community’s subgraphs.

The Walk Trap Algorithm [36] allowed us to identify 32 communities within Odyssey’s social network (see Fig 1 coloring of vertices). We have run this algorithm using The R Project for Statistical Computing [47]. Particularly, we have employed the Igraph R Package [48] using the default setting of the walktrap.community function. Studying the topological properties of each community, we found a strong fit for max degree of each community (Fig 4). We could identify that this max degree ranking fits a power-law distribution (R^2^ = 0.98) which can be a way to infer that the leader (*i*. *e*., a character with a higher degree) of each community relates to each other in a special manner. This histogram follows the power law function

$$k_{max}(r)=\alpha .r^{-\gamma },$$ (6)

where *r* stands for the community’s ranking, ranging from 1 to 32. The parameters of the fitting displayed in Fig 4 are *α* = 249.3 and *γ* = 1.503. We interpret this power-law decay of the communities’ max degree as a consequence of how the communities participate in related events. The building criteria of the network indicate to us that communities are strongly related by events that synthesizes them. Roughly speaking, we can consider the leader of each community as the vertex that synthesizes the whole community. This claim is reasonable since the leaders of the communities’ participate in all or at least in the majority of the events which comprises such communities’. In other words, the leaders are excellent representatives of communities and are related to each other in power-law pattern, as described in Fig 4. This particular distribution, in the case of the representatives (*i*.*e*., leaders) of each community, just means that there are few important communities (*i*. *e*., high max degree) and many secondary communities. In addition, this effect does also indicate to us that most relevant communities are related to each other, and tends to connect mainly between themselves.

**Fig 5:**
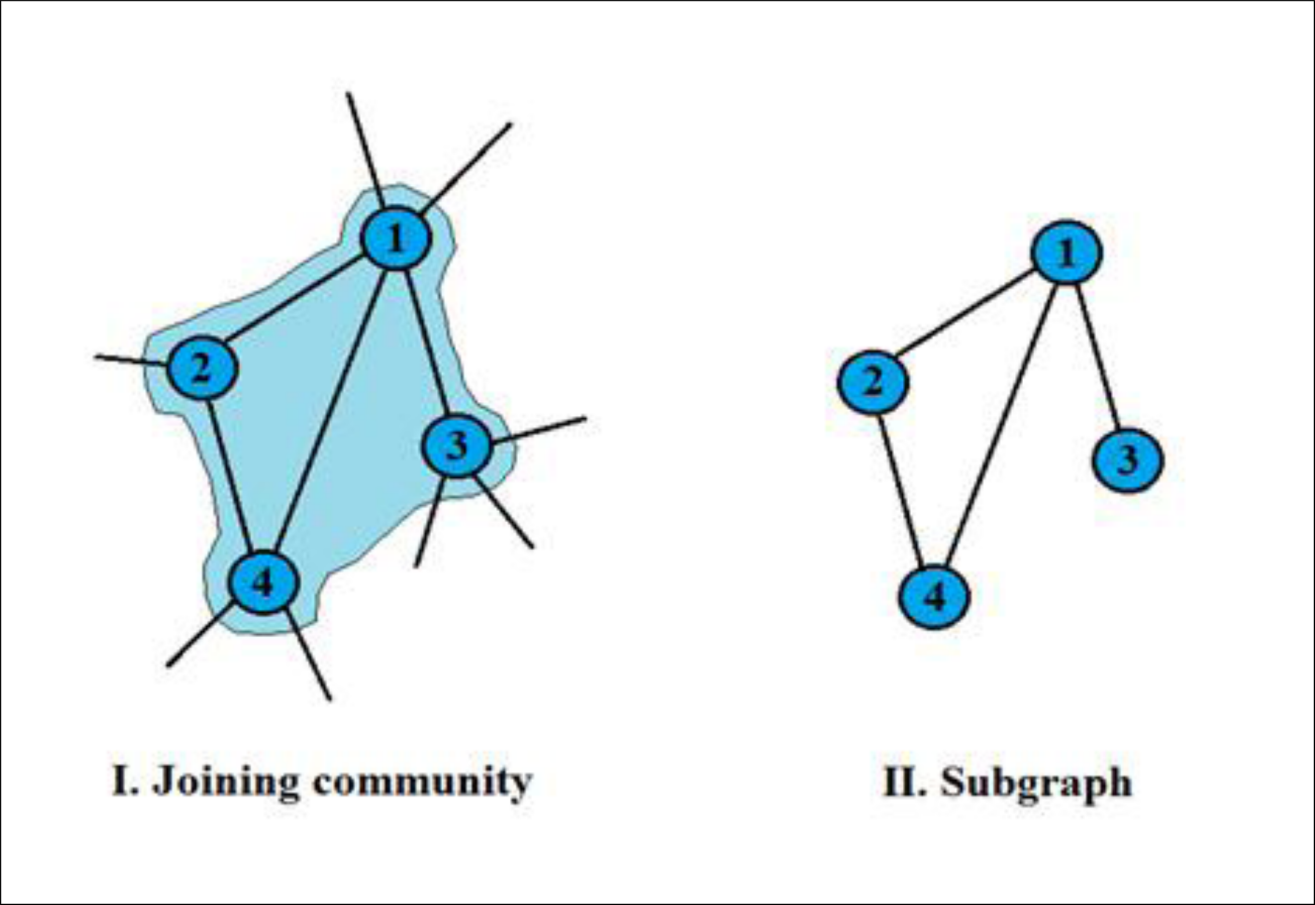
Community concepts. I) Joining community: the nodes keep their degree and topological dependences with the rest of the network; II) Subgraph: the topological quantities depends only on the community alone.

We’ve individualized the most influential communities by the criterion N ≥ 10 (*i*.*e*, number of vertices), and calculated their topological measures (Table 4). To calculate topological quantities to each community, we have treated them in two ways: one that we call joining communities, which the community is analyzed as part of the network; and other the subgraph, which is the disaggregation of the community from the network. An explanatory scheme can be found in Fig 5 to avoid misunderstanding. This differentiation is very important since topological properties depend on it.

**Table 4:** Statistical properties of communities.

*Community*	*Type*	*N*	*k*_*max*_	〈*k*〉	*ℓ*	*ℓ*_*rand*_	*C*	*C*_*rand*_	*r*
*Com*. *A*	*joining*	*83*	*214*	*8*.*30*	*2*.*58*	*2*.*75*	*0*.*7*	*0*.*15*	*-0*.*15*
	*subgraph*	*83*	*66*	*8*.*28*	*2*.*11*	*2*.*44*	*0*.*4*	*0*.*23*	*-0*.*29*
*Com*. *B*	*joining*	*42*	*52*	*9*.*10*	*2*.*02*	*2*.*09*	*0*.*67*	*0*.*26*	*-0*.*15*
	*subgraph*	*42*	*29*	*6*.*14*	*2*.*14*	*2*.*02*	*0*.*64*	*0*.*42*	*-0*.*24*
*Com*. *C*	*joining*	*73*	*76*	*13*.*4*	*2*.*05*	*2*.*30*	*0*.*61*	*0*.*22*	*-0*.*15*
	*subgraph*	*73*	*46*	*11*.*0*	*2*.*12*	*2*.*00*	*0*.*59*	*0*.*54*	*-0*.*28*
*Com*. *D*	*joining*	*10*	*10*	*3*.*90*	*1*.*86*	*2*.*64*	*0*.*66*	*0*.*19*	*-0*.*15*
	*subgraph*	*10*	*6*	*2*.*20*	*2*.*00*	*1*.*94*	*0*.*28*	*0*.*34*	*-0*.*40*
*Com*. *E*	*joining*	*11*	*19*	*9*.*81*	*1*.*30*	*1*.*85*	*0*.*65*	*0*.*26*	*-0*.*15*
	*subgraph*	*11*	*9*	*6*.*18*	*1*.*30*	*1*.*30*	*0*.*70*	*0*.*70*	*-0*.*23*
*Com*. *F*	*joining*	*25*	*28*	*9*.*60*	*1*.*78*	*2*.*19*	*0*.*66*	*0*.*33*	*-0*.*15*
	*subgraph*	*25*	*19*	*6*.*88*	*1*.*82*	*1*.*77*	*0*.*66*	*0*.*55*	*-0*.*26*
*Com*. *G*	*joining*	*20*	*33*	*1*.*9*	*1*.*31*	*2*.*00*	*0*.*74*	*0*.*18*	*-0*.*15*
	*subgraph*	*20*	*16*	*12*.*5*	*1*.*31*	*1*.*31*	*0*.*82*	*0*.*82*	*-0*.*12*
*Com*. *H*	*joining*	*13*	*14*	*14*.*0*	*1*.*00*	*1*.*00*	*1*.*00*	*1*.*00*	*-0*.*15*
	*subgraph*	*13*	*12*	*12*.*0*	*1*.*00*	*1*.*00*	*1*.*00*	*1*.*00*	*-0*.*01*

Type (joining community or subgraph), size (*N*), maximum degree (*k*_max_), mean degree 〈*k*〉, average path length (*ℓ*), average path length for a randomly created community (*ℓ*_*rand*_), clustering coefficient (*C*), clustering coefficient for a randomly created community (C_rand_) and assortativity (*r*)

To aid on our analysis, we have composed the Tables 5 and 6, which comprises the behavior of network properties as the communities are attached to one another in a specific order. We choose to attach communities in two ways: one from the most relevant community, which is Community A to the lesser communities (Table 5) and another by beginning with the lesser communities until Community A. This procedure allows us to see how and when network properties arise via community linking (Table 6).

**Table 5:** Adding up communities’ from above.

***Property***	***A***	***+B***	***+C***	**+D**	***+E***	***+F***	***+G***	***+H***	***+R***
***N***	83	156	198	223	243	256	267	277	342
***E***	334	862	1088	1240	1414	1518	1588	1616	1747
**〈*k*〉**	8.28	11.05	10.98	11.12	11.63	11.85	11.89	11.66	10.21
***ℓ***_***max***_	4	4	4	4	4	4	5	5	6
***ℓ***	2.11	2.23	2.29	2.31	2.33	2.31	2.34	2.35	2.58
***ℓ***_***rand***_	2.44	2.55	2.57	2.63	2.56	2.59	2.61	2.60	2.75
***C***	0.40	0.37	0,32	0,30	0,31	0,31	0,31	0.30	0.28
***C***_***rand***_	0.23	0.19	0.17	0.15	0.14	0.13	0.12	0.12	0.11
***G***	1.00	1.00	1.00	1.00	1.00	1.00	1.00	1.00	1.00
***r***	-0.29	-0.24	-0.22	-0.19	-0.17	-0.16	-0.16	-0.16	-0.15

The behavior of statistical properties of the network by the attachment of communities: from community *A* to *R*. In which *R* stands for the set of all remaining lesser communities.

**Table 6:** Adding up communities’ from below.

***Property***	***R***	***+H***	***+G***	**+F**	***+E***	***+D***	***+C***	***+B***	***+A***
***N***	65	75	86	99	119	144	186	259	342
***E***	67	78	116	194	319	407	563	1026	1747
**〈*k*〉**	2.06	2.08	2.69	3.92	5.36	5.65	6.05	7.92	10.21
***ℓ***_***max***_	4	4	7	7	7	7	12	9	6
***ℓ***	1.79	1.90	2.80	2.55	2.25	2.27	4.01	3.47	2.58
***ℓ***_***rand***_	5.56	4.85	4.10	3.39	3.20	3.10	3.15	2.97	2.75
***C***	0.72	0.65	0.70	0.90	0.90	0.83	0.67	0.50	0.28
***C***_***rand***_	0.02	0.02	0.04	0.12	0.12	0.70	0.09	0.10	0.11
***G***	0.20	0.17	0.30	0.26	0.21	0.20	0.61	0.90	1.00
***r***	0.54	0.41	0.64	0.90	0.84	0.63	0.05	-0.03	-0.15

The behavior of statistical properties of the network by the attachment of communities: from community *R* to *A*.

Inspecting Table 4 we can see that all communities are individually disassortative. Moreover, disassortativity is constant when we take Community A as a base for the attachment of other communities (Table 5). We would also like to point it out that from this perspective, assortativity increases as we proceed from A to R. In the other hand, positive assortativity (that signals real network) is observed on the whole network when we disregard Communties’ A and B (mostly A) (Table 6). A similar effect happens to the size of giant component: it is ≤ 90% until the attachment of Community A (Table 6). This means that com. A is responsible for a larger giant component. In other words, it makes the network denser. This statement is confirmed if we look at the behavior of the size of the giant component in Table 5: it will never be less than 100% since Community A keeps all up-coming vertices from attachment into the giant component. In this way, we can imagine Community A as a “backbone” structure which all vertices tend to attach to.

Furthermore, “small-worldness” (*ℓ* ≈ *ℓ*_*rand*_ and *C* ≫ *C*_*rand*_) is only observed when Community A is attached lastly (Table 6); so we can consider it as the community most responsible for the introduction of short-cuts throughout the network. Moreover, if we look at the behavior of the differences Δ*ℓ* = |*ℓ* − *ℓ*_*rand*_| and ΔC = |*C* − *C*_*rand*_|in the Table 5, we can note that they do not diverge as in the Table 6. This is a fact since Community A is the base for attachment in Table 5, and contains most of the short-cuts present in the whole network will not differ much as other communities attach to A.

Until now, the results from the Tables 4, 5 and 6, indicate that Community A seems to have a special role in Odyssey and in its global properties. For now, let us recall that we have shown that the network’s degree distribution is best described by a power-law with an exponential cut-off, and we’ve proposed to explain it by means of communities.

**Fig 6:**
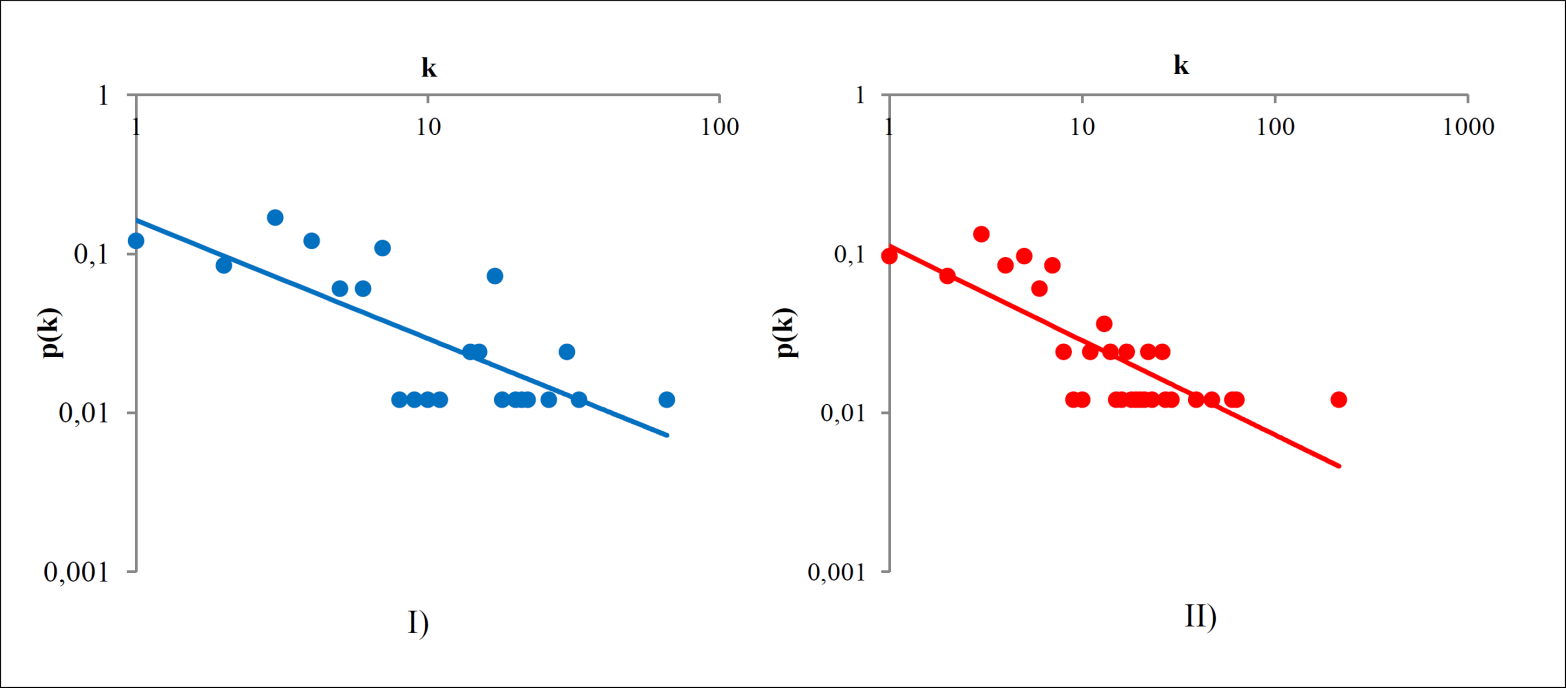
Degree distribution of community A. I) Subgraph aspect with the blue line as the power law fit with *R*^2^ = 0.55, and II) Joining graph aspect with the red line as the power law fit with *R*^2^ = 0.61.

**Fig 7:**
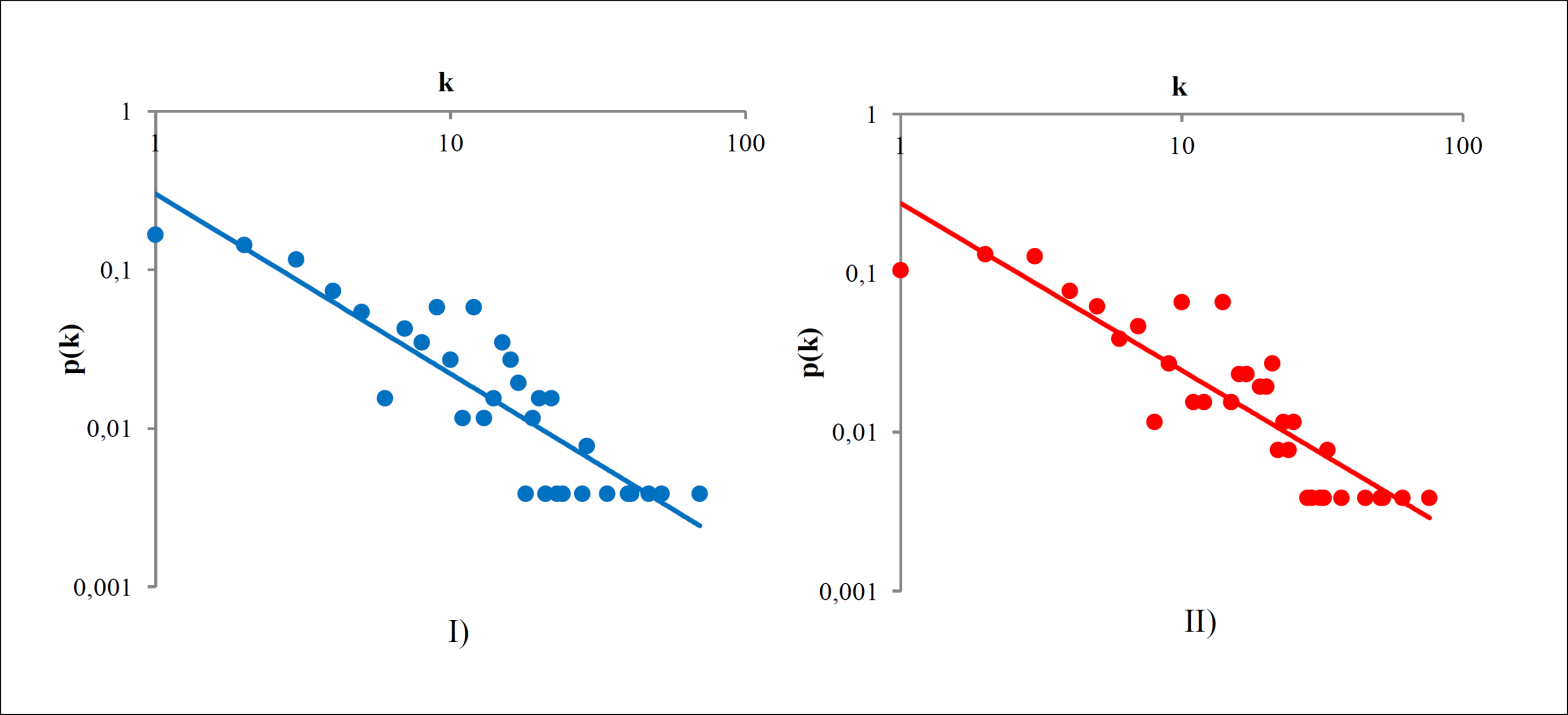
Degree distribution of Odyssey disregarding community A. I) Subgraph aspect with the blue line as the power law fit with *R*^2^ = 0.79, and II) Joining graph aspect with the red line as the power law fit with *R*^2^ = 0.79.

To begin to understand this feature, we look at the character composition of Community A (Table 7). With little knowledge of Odyssey, one may conclude that this community is mainly composed of the mythological characters such as gods, heroes, and monsters. In addition, we also have seen that the removal of central mythological characters leads the network to resemble a real social network, “fixing” its giant component size and disassortativity (Table 3). These two propositions induce us to plot the degree distribution of Community A, and then to plot the degree distribution of Odyssey disregarding A. These plots can be found in Figs 6 and 7, respectively. We have considered them as joining graph and subgraph, and also tested their fit for the power-law and exponential law distributions.

**Table 7:** Character’s composition of each eight most relevant community and the remaining 24 communities.

Community	Character’s composition
Com. A	Odysseus, Zeus, Hera, Hades, Hestia, Demeter, Apollo, Ares, Artemis, Athena, Hermes, Hephaestus, Dionysus, Calypso, Kronos, Poseidon, Antiphus, Hebe, Amphitrite, Zephyrus. Rhadamanthus, Aphrodite, Aurora, Tithonus, Jason, Euro, Noto, Nausitoo, Nausicaa, Latona, Eurimedusa, Arete, Peribea, Eurimedonte, Rexenor, Erechtheus, Aecheneus, Pontonous, Tithius, Demodocus, Aeolus, Persephone, Pelias, Alcmene, Heracles, Megara, Chloris, Leda, Iphimedeia, Otho, Orion, Leto, Phaedra, Procris, Ariadne, Minos, Mera, Climena, Erifila, Tiresias, Tantalus, Gorgon, Kreanaiai, Limnatides, Pegaiai, Potameides, Atlas, Boreas, Terra, Eurito, Hippotes, Anfitrion, Creontes, Aloeus, Efialto, Theseus, Memnon, Sisyphus, Piritoo, Eleionomae, Phidon.
Com. B	Orestes, Aegisthus, Menelaus, Nestor, Agamemnon, Achilles, Ajax, Patroclus, Antilochus, Atreus, Diomedes, Philoctetes, Idometius, Fhaebus, Hermione, Helen, Adraste, Alcippe, Asfalion, Anticlus, Tidida, Idotea, Proteus, Arena, Tiestes, Fedimo, Priamus, Aepeus, Kassandra, Clytemnestra, Peleus, Euripilus, Tideus, Peias, Neoptolemos, Philus, Delfobo, Philomelidae, Eacus, Telephus, Telaman, Orsilochus.
Com. C	Telemachus, Mentes, Antinous, Eurymachus, Phemius, Laertes, Penelope, Eurycleia, Egipcius, Eurynomo, Pisenor, Ikarios, Thetis, Halitherses, Mentor, Liocritus, Noemone, Medonte, Dolios, Arcesius, Litima, Eumelus, Polybius, Anticleia, Eumaeus, Shepherd 1, Shepherd 2, Shepherd 3, Shepherd 4, Theoclymenus, Piraeus, Amphinomus, Antinomus, Nisus, Amphius, Melanthius, Phormius, Eurynome, Antonoa, Hippodamia, Euridamante, Pisantro, Melantho, Mulius, Antolichus, Philetius, Cresipus, Agelaus, Liodes, Amphimedonte, Demoptolemus, Euriades, Elatus, Polinus, Leocritus, Eutypes, Ops, Mycenae, Mastor, Evenor, Mesaulius, Crimena, Clitius, Iro, Aechetus, Icmalius, Eurynomia, Damastor, Aenopo, Politherses.
Com. D	Antiopa, Amphione, Cromius, Son of Pandareu, Zeto, Iaso, Periclymenus, Pero, Pandareu.
Com. E	Peisistratus, Traedimedes, Neleus, Echefrone, Estratius, Perseus, Aretus, Eurydice, Policasta, Aeteoneus, Climeno.
Com. F	Baius, Eurylochus, Perimedes, Helios, Polyphemus, Lotophagus, Circe, Ecta, Perse, Polites, Underling 1, Underling 2, Underling 3, Underling 4, Elpenor, Erebus, Crateide, Faetusa, Neera, Hyperion, Oceanus, Scylla, Lampétia, Charybdis.
Com. G	Alcinous, Laodamas, Amphialus, Euryalus, Halius, Clytoneus, Acroneus, Acialo, Elatreus, Nauteus, Prymneus, Prymreus, Anchyalus, Ponteus, Proreus, Toone, Anabeesineus, Eretmeus, Polineus, Naubolus.
Com. H	Aeolus’s Wife, Aeolus’s Son 1, Aeolus’s Son 2, Aeolus’s Son 3, Aeolus’s Son 4, Aeolus’s Son 5, Aeolus’s Son 6, Aeolus’s Daughter 1, Aeolus’s Daughter 2, Aeolus’s Daughter 3, Aeolus’s Daughter 4, Aeolus’s Daughter 5, Aeolus’s Daughter 6.
Remaining	Iphitus, Aeuritus, Salmoneus, Tiro, Creteus, Aesone, Pherete, Amitaone, Frontis, Anaetor, Melampus, Philachus, Phorcis, Theosa, Aecles, Amphiraus, Alcmeon, Amphilochus, Thelemus, Eurymides, Diocles, Ortilochus, Alpheus, Tan, Pean, Polydamnas, Leucotey, Epicasta, Cadmo, Edipus, Lestrogony’s Explorer 1, Lestrigony’s Explorer 2, Herald of Lestrigony, Antiphates, Antiphates’s Wife, Antiphates’s Daughter, Megapentis, Aelector, Aelector’s Daughter, Mantius, Poliphides, Clitus, Tindaro, Castor, Polux, Ithachus, Netitus, Polictor, Maraon, Evanteus, Thisiphone, Megera, Alectus, Cresius, Ormenius, Phenicia, Aribante, Thoante, Andremone, Eurytion, Piritous, Phronius, Boetus, Terpias.

**Fig 8:**
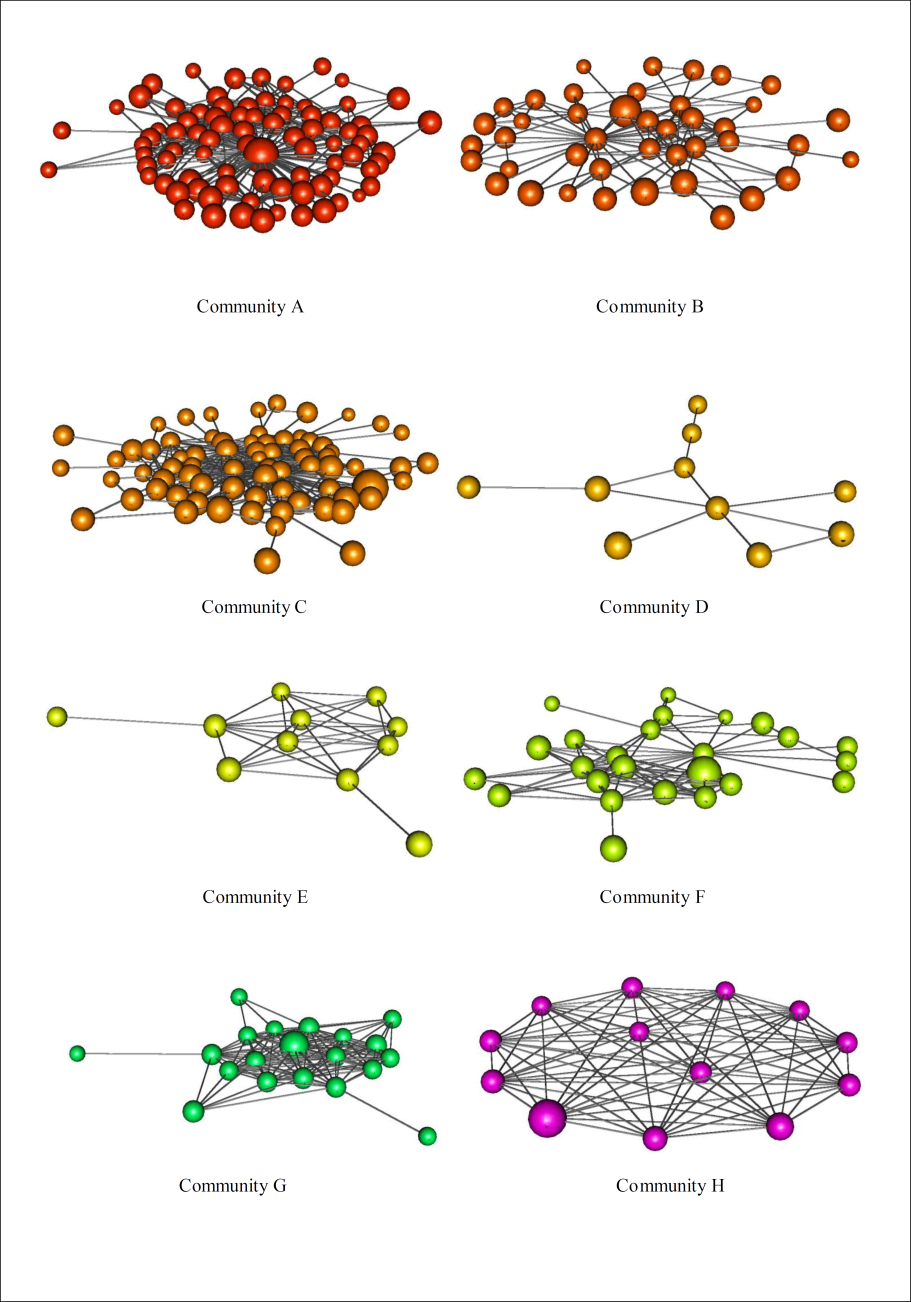
The eight most relevant communities of the Odyssey’s network. Communities: A) God’s Assembly, B) Troy’s War, C) Ithaca’s Events, D) Secondary Myths, E) Nestor’s Relatives, F) Odysseus Journey’s Events, G) Phoenician’s Island and H) Aeolus’s Island.

- The values of squared Pearson coefficient for the power-law fit are displayed on the label of Figs 7 and 8. Thus, we have only displayed the power-law fit, since that in all cases it was the best fit. The values of squared Pearson coefficients for the exponential law are: Community A as subgraph: *R*^2^ = 0.30.
- Community A as joining graph: *R*^2^ = 0.14.
- Odyssey network disregarding A as subgraph: *R*^2^ = 0.59.
- Odyssey network disregarding A as joining graph: *R*^2^ = 0.66.

Studying these results, we note that Odyssey without Community A is strongly scale-free, both as a subgraph and a joining graph since their fit is *R*^2^ = 0.79. This value indicates two important features. First, the “scale-freeness” of Odyssey is independent of Community A as we note that *R*^2^ is equal in the subgraph and joining graph cases; and second, the power law with exponential cut-off for the whole Odyssey is observed because Community A is the most responsible for the exponential part and the rest for the power law part. We would like to emphasize once again that Community A is composed by high connected nodes that represents mythological characters. This implies that the exponential “tail” of the degree distribution is explained by the presence of a set of high connected mythological characters that drags the network to a fictional type of network.

Besides Community A has a better fit for the power law (*R*^2^ = 0.55 as subgraph and *R*^2^ = 0.61 as joining graph), the exponential effect comes into play when we connect A to the rest of the network. This means that the edges between A and the rest of Odyssey is the cause of this feature. We can confirm this observing by the increase on *R*^2^ from subgraph to joining graph in the power-law case; and when we observe a decrease on *R*^2^ from subgraph to joining in the exponential law case. In other words, when A is attached to the rest of the Odyssey; it becomes “more” exponential and “less” power-law. We assume that the rest of Odyssey is dragging A to a power law distribution. Corroborating to this statement, we can look at the increase of *R*^2^ for the exponential law in the rest of Odyssey, but still, its *R*^2^ for power-law is unaffected. This concludes the explanation for the power law with exponential cut-off for the Odyssey’s network. Now we proceed to explain the lack of hierarchy of it.

As we know, hierarchical networks are characterized by a network composed of small groups of vertices organized in a hierarchical manner which composes scaling larger groups. According to Ravasz and Barabási [11], most real networks are scale-free and display high clustering coefficient, and these features are a consequence of a hierarchical organization. The common way to test it is to find a significant fit for a power-law distribution for Eq. (5). We argue that in the case of Odyssey, there are no small groups of vertices that compose larger groups, but there are larger groups that compose an even larger group. We mean that communities are larger groups that compose Odyssey by the rule displayed in Fig 4. So we cannot actually affirm that the network is hierarchical in the Ravasz and Barabási sense, but we can affirm that the communities are related to each other in a hierarchical way, based on its leader (*i*. *e*., vertex with maximum degree). Nevertheless, Odyssey displays a scale-free part and can be considered as highly connected which implies that it can still be considered as hierarchical since these two criteria are the cause of the distribution expected in Eq. (5).

Besides identifying communities in graph theory sense, the community detection method has also shown that the communities have social meaning. That is, if a set of characters belongs to a community, it means that these characters participate in a related set of events of Odyssey. For example, Community A contains all the elements of the chants concerning the “Assembly of the gods”; the Community B is composed by the most remarkable heroes that fought on the Trojan War; Community C stands for the events on Ithaca. Community D is composed of secondary characters like nymphs, godlike monsters, and minor gods; Community E takes into account the sons and daughters of Nestor, capturing the episode where Telemachus search his father; Community F ensembles several epics scenarios of Odysseus’s Journey, capturing events such as: Lotofagus’s Island, Circe’s Lair, Hyperion Sun’s Island and Scila and Caribdis episode. This community also contains most of the main journey’s comrades of Odysseus; Community G represents the Odysseus passage to the land of Phaeacians; and finally Community H, which stands for Odysseus’s visit to Aeolus’s Island, so its characters are composed mainly by Aeolus sons and daughters.

**Fig 9:**
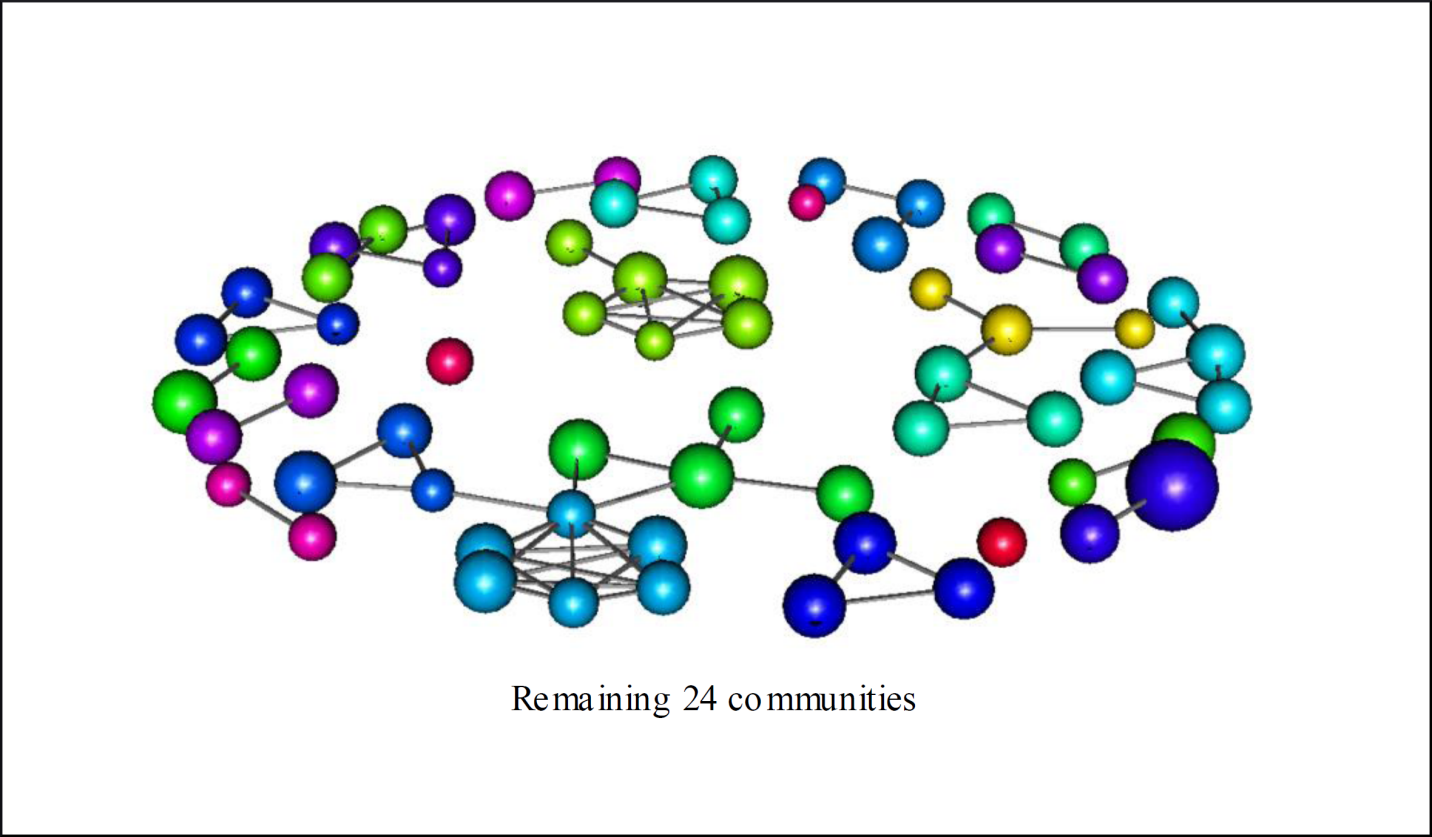
The other 24 less relevant communities of Odyssey’s network.

All eight most relevant communities are displayed for appreciation in Fig 8 while the remaining of the communities can be found in Fig 9. We emphasize that the display of such communities’ is relevant in order to give a visual idea of how they are structured. In addition, we also have provided the character composition of each community (Table 7). Looking at these results we can observe a superposition between related characters based on the story and the vertices that compose communities. This reflects a strong relationship between characters composition of communities and the criteria for the building process of the network. If we recall, our criteria are based mainly on two social interactions (we are considering the conversational and indirect criteria essentially the same): directly and by means of important events. Still, we can see direct interactions as minor events, such as the case that Odysseus has a colloquium with other characters.

Overall, minor events condense a few characters and important events condense many characters. With this in mind, we can interpret a community as a set of strong interdependent events that concentrate on close related characters, while relations between communities are secondary dependent events which do not necessarily demands characters concentration. By the characteristics of events, we can roughly say that minor events connect communities while important events define communities.

We have seen that there is also a special relationship between communities: a power law for the ranking of the maximum degree of each community. In social event terms, this means that the events played on important communities determined the events that happen in lesser communities. The narrative structure corroborates this assumption since, for instance, Community A stands for the Assembly of Gods which decides the penalty and also the redemption of Odysseus. The same can be observed in Communities B and C since they represent the set of most influential events in which the most relevant characters participate. This sort of relationship between communities determines a sort of hierarchy of events. Conversely, we defend that important events are condensed on communities and this implies that communities are associated hierarchically.

The topological results have also shown that Community A has a special role on Odyssey. It drags the network to fictional aspects while maintaining important short-cuts between unrelated sets of import events. Degree distribution becomes semi exponential, the giant component becomes 100% and disassortativity increases. We attribute all these features to the fact that Community A is composed almost purely of god, heroes, and monsters. And as shown in Table 3, this sort of characters drags the network toward fictional nature. Finally, we can look at this community and say that it is, by itself, a fictional network. Additionally, when we disregard community from Odyssey, the resulting network has the most important features for a real social network. It is strongly scale-free, it has giant component equals 90%, is weakly disassortative, highly clustered, vulnerable to target attacks while resilient to random attacks.

The analysis of community allowed us to describe Odyssey’s statistical properties in a more accurate way. Allowing us to determine when and how some properties emerge, such as the power law with exponential cut-off and the lack of hierarchy between vertices. It also provided us a way to find another form of hierarchy, such as the maximum degree ranking that is explained by a power law distribution. Beyond statistical, this method also has shown us that the nature of social network based on narratives is strongly dependent on the sort of characters that play a role on them.

## Conclusions

The statistical analysis demonstrated that Odyssey’s social network is small world, not hierarchical, and semi scale-free. The degree distribution follows a power-law with an exponential cut-off. It is also not highly clustered, but resilient to random attacks and vulnerable to target attack. Additionally, it has giant component’s size bigger than 90% and is disassortative. The removal of important mythological characters implied in a reduction of the giant component and in an increase in assortativity. This procedure indicates that the presence of mythological characters causes the network to be fictional.

The analysis of communities allowed us to understand the meaning of a degree distribution given by a power law with exponential cut-off. The community A, that represents the set of events most relevant in Odyssey, causes the degree distribution to be exponential since it is mostly composed of mythological characters. And the lack of hierarchy between vertices was explained by another sort of hierarchy: one that is dependent on the maximum degree of each community. This implies that there is a hierarchy between large groups which composed the final network, these large groups happens to be the communities. Besides confirming topological measures and describing how and when they emerge, community analysis showed that the character composition of communities is strongly related to their participation on Odyssey. This means that communities stand for well-defined sets of events that define them.

Finally, we conclude that Odyssey might be an amalgam of real based societies and mythological content organized in communities. This work has a strong appeal for its new community analysis and also for confirming Carron and Kenna results about universal properties of mythological networks.
